# Transactivation of ErbB Family of Receptor Tyrosine Kinases Is Inhibited by Angiotensin-(1-7) via Its Mas Receptor

**DOI:** 10.1371/journal.pone.0141657

**Published:** 2015-11-04

**Authors:** Saghir Akhtar, Bindu Chandrasekhar, Sreeja Attur, Gursev S. Dhaunsi, Mariam H. M. Yousif, Ibrahim F. Benter

**Affiliations:** 1 Department of Pharmacology and Toxicology, Faculty of Medicine, Kuwait University, Safat 13110, Kuwait; 2 Department of Pediatrics, Faculty of Medicine, Kuwait University, Safat 13110, Kuwait; INSERM, FRANCE

## Abstract

Transactivation of the epidermal growth factor receptor (EGFR or ErbB) family members, namely EGFR and ErbB2, appears important in the development of diabetes-induced vascular dysfunction. Angiotensin-(1–7) [Ang-(1–7)] can prevent the development of hyperglycemia-induced vascular complications partly through inhibiting EGFR transactivation. Here, we investigated whether Ang-(1–7) can inhibit transactivation of ErbB2 as well as other ErbB receptors in vivo and in vitro. Streptozotocin-induced diabetic rats were chronically treated with Ang-(1–7) or AG825, a selective ErbB2 inhibitor, for 4 weeks and mechanistic studies performed in the isolated mesenteric vasculature bed as well as in cultured vascular smooth muscle cells (VSMCs). Ang-(1–7) or AG825 treatment inhibited diabetes-induced phosphorylation of ErbB2 receptor at tyrosine residues Y1221/22, Y1248, Y877, as well as downstream signaling via ERK1/2, p38 MAPK, ROCK, eNOS and IkB-α in the mesenteric vascular bed. In VSMCs cultured in high glucose (25 mM), Ang-(1–7) inhibited src-dependent ErbB2 transactivation that was opposed by the selective Mas receptor antagonist, D-Pro^7^-Ang-(1–7). Ang-(1–7) via Mas receptor also inhibited both Angiotensin II- and noradrenaline/norephinephrine-induced transactivation of ErbB2 and/or EGFR receptors. Further, hyperglycemia-induced transactivation of ErbB3 and ErbB4 receptors could be attenuated by Ang-(1–7) that could be prevented by D-Pro^7^-Ang-(1–7) in VSMC. These data suggest that Ang-(1–7) via its Mas receptor acts as a pan-ErbB inhibitor and might represent a novel general mechanism by which Ang-(1–7) exerts its beneficial effects in many disease states including diabetes-induced vascular complications.

## Introduction

The precise mechanisms underlying the development of diabetes-induced vascular complications such as altered vascular reactivity, hypertrophy and dysfunction are poorly understood and may involve diverse multiple signaling pathways that are affected by hyperglycemia [1,2,3]. Emerging evidence suggests that dysregulation of the epidermal growth factor (EGF) receptor (EGFR or ErbB) family of receptor tyrosine kinases (RTKs) appears important in mediating hyperglycemia-induced vascular dysfunction [4–11].

The ErbB family of RTKs that act as central hubs or signal relays for diverse pathways are important regulators of cellular functions such as growth, proliferation, differentiation, motility, invasiness and apoptosis [6, 12, 13]. This family comprises four members: ErbB1 through ErbB4 (or HER1 through HER4), that upon binding with an appropriate ligand (e.g. EGF) induce phosphorylation of specific tyrosine residues within the intracellular kinase domain of the receptor and this results in either homo- or heterodimerization amongst family members. Dimerization of ErbBs leads to activation of multiple downstream signalling pathways such as the mitogenic Ras/Raf/ extracellular-signal-regulated kinase 1/2 (ERK1/2), the p38 mitogen activated protein (MAP) kinase or the PI3-kinase/Akt survival pathways [12– 14]. The best characterized member of the family is ErbB1, better known as EGFR1 or simply EGFR, and along with ErbB4 is an intact receptor with a ligand binding domains as well a functional intracellular tyrosine kinase. The ErbB2 receptor lacks a known ligand and relies on heterodimerization with other family members for signaling whereas ErbB3 lacks an active kinase [12, 14]. Transactivation of ErbBs can also occur via G-protein coupled receptors (GPCRs), such as angiotensin II (Ang II), thrombin, aldosterone, endothelin and norephinephrine (NE) [5,15–19], by mechanisms that involve upstream non-receptor tyrosine kinases such as c-src [5] and/or mediated via metalloprotease and/or ADAM(a disintegrin and metalloprotease)-dependent shedding of cell-surface bound EGF-like ligands [15,17].

We have previously shown the diabetes and/or hyperglycemia induces upregulation of EGFR and ErbB2 expression and phosphorylation that leads to vascular dysfunction via pathways involving ERK1/2, p38 MAPK and ROCKs [4–5]. Pharmacological antagonism with selective inhibitors of either EGFR phosphorylation by AG1478 or ErbB2 receptor phosphorylation by AG825 corrected the vascular dysfunction associated with diabetes as evidenced by a normalization of the hyper-responsiveness of blood vessels to vasoactive agents such as Ang II and Norephinephrine (NE) [4,7,8,20]. Indeed, upregulation of signaling via the octapeptide Ang II (a major player in Renin-Angiotensin System (RAS) or NE (via the GPCR, α_1_-adrenoceptor) might involve cross-talk with EGFR/ErbB family of receptors [5,15,19].

The RAS is comprised of two main counter-regulatory axes [21,22]. The ACE-Ang II-AT1 receptor axis is detrimental to vascular function in diabetes where it mediates vasoconstriction, oxidative stress and pro-inflammatory signaling [23]. In contrast, the counter-regulatory ACE2-Ang-(1–7)- MasR axis, where the heptapeptide Ang-(1–7) is the main effector is beneficial to vascular function largely by opposing the detrimental effects of Ang II [21– 25]. Thus, Angiotensin-(1–7) [Ang-(1–7)], which is a metabolite of Ang II, exhibits antihypertensive, antithrombotic and antiproliferative properties [23–24, 26–28]. We have previously shown that Ang-1-7 can prevent vascular dysfunction in models of diabetes without markedly correcting hyperglycemia [28–29]. However, its exact mechanisms of action in the diabetic vasculature are not known.

In a rat model of type 1 diabetes, we previously showed that Ang-1-7 is an inhibitor of EGFR tyrosine kinase activity and resultant downstream signaling via ERK1/2 and p38 MAP kinase [5] that might help explain its beneficial actions in the diabetic vasculature. However, its effects on ErbB2, or other members of ErbB/EGFR family of receptor tyrosine kinases, are not known. Here we sought to determine whether Ang-(1–7) is an inhibitor of ErbB2 transactivation arising from various triggers and whether it had any effect on the phosphorylation of other ErbBs in the vasculature of type 1 diabetes and in cultured vascular smooth muscle cells (VSMC). Thus, we showed that Ang-(1–7 inhibits ErbB2 transactivation induced by diabetes or high glucose as well as Ang II- or NE-mediated G-protein coupled receptor activation. Further we showed that diabetes- or glucose-induced ErbB3, and Erbb4 transactivation could be attenuated by Ang-(1–7) treatment thereby implying that Ang-(1–7) is a pan-inhibitor of the ErbB family of receptor tyrosine kinases.

## Methods

### Drugs

AG825 (((E)-3-[3-[2-Benzothiazolythio)methyl]-4-hydroxy-5-methoxyphenyl]-2-cyano-2-propen-amide)) and AG1478 (N-(3-chlorophenyl)-6,7-dimethoxy-4-quinazolinanine hydrochloride) was purchased from Tocris (Bristol, UK). Streptozotocin (STZ), SU6656 ({2,3-dihydro-N, N-dimethyl-2-oxo-3- [{4,5,6,7 -tetrahydro-1H- indol-2-yl}methylene]-1H-indole-5-sulphonamide), Losartan, Prazosin and Ang-(1–7) were all purchased from Sigma Chemical Co. (St Louis, MO, USA). D-Pro^7^-Ang-(1–7) was purchased from American Peptide Company (Sunnyvale, CA, USA).

### In vivo studies

Male Wistar rats weighing approximately 250 g were used in this study as per the following groups (n = 7 per group). Group 1: non-diabetic (Control) animals; Group 2: STZ (55 mg·kg^–1^ body weight)-treated animals; Group 3: STZ + Ang-(1–7) (576 μg kg^–1^ day^–1^ i.p.). Group 4: STZ + AG825 (1 mg kg^–1^ i.p alt diem). Additional animals in Groups 1 and 2 (n = 7) were also used for acute, ex-vivo, drug treatments of the perfused isolated mesenteric bed with Ang-(1–7) for the signalling studies (n = 7). A total of 42 animals were used in this study. All animal care and experimental procedures were conducted in accordance with the National Institutes of Health Guide for the Care and Use of Laboratory Animals (NIH Publication no. 85–23, Revised 1985) and were approved by Kuwait University Research Sector/Administration’s Health Sciences Research Ethics Committee following consideration of both the scientific and ethical aspects of the study design.

### Induction of diabetes and treatment regimens

Diabetes was induced by a single i.p. injection of 55 mg·kg–1 body weight STZ dissolved in citrate buffer (pH 4.5) as described by us previously [4]. Age-matched control rats were injected with the citrate buffer vehicle used to dissolve STZ. Body weight and basal glucose levels were determined before the STZ injection, using an automated blood glucose analyzer (Glucometer Elite XL). Blood glucose concentrations were determined 48 h after STZ injection. Rats with a blood glucose concentration above 250 mg·dL^–1^ were declared diabetic. The animals’ body weights and the diabetic state were re-assessed after 4 weeks prior to sacrifice. The regimen for in vivo drug administration for AG825 and Ang-(1–7) was based on our previous studies in models of diabetes and/or hypertension [4–5, 7, 24, 28–30]. In all cases, the last dose of the pharmacological agents was administered at 4 pm the day before and animals were sacrificed by cervical dislocation at 9 am the next day. This strategy leads to clinically relevant circulating levels of Ang- (1–7) in rats that are about 25-fold higher than basal [30]. For the acute (ex vivo) studies, isolated mesenteric vascular bed was incubated with 1 micromolar Ang-(1–7) in Krebs-Henseleit buffer solution for 1h in a shaking water bath at 37°C, thereafter snap frozen in liquid nitrogen prior to storage at -80°C and subsequent signaling studies by Western blotting as described below.

### Western blotting studies

Western blotting for total or phosphorylated forms of ErbB2, EGFR, Src, ERK1/2, p38 MAPK, ROCK2, eNOS and IkB-α was performed essentially as described by us previously [4–5, 7–8, 31]. Briefly, rat mesenteric vascular beds were isolated, snap-frozen in liquid nitrogen and stored at -80°C. The tissue samples were defrosted in ice then lysed as described previously [31]. Lysates were then centrifuged at 16 000g for 20 min at 4°C and supernatants were collected and protein concentration estimated by Bio-Rad BCA protein assay (Hercules, CA, USA). Aliquots containing equal amounts of protein were subjected to SDS-PAGE and transferred onto nitrocellulose membrane (Schleicher & Schuell, Dassel, Germany). Membranes were then incubated with either monoclonal antibodies (Cell Signaling, Danvers, MA, USA) to detect phosphorylated and total forms of ErbB2, EGFR, ErbB3, ErbB4 (all ErbB bands seen at approximately 175–180 kDa), Src (at approx. 60 kDa), ERK1/2 (at 42/44 kDa), p38 MAPK (at 38 kDa), ROCK 2 ((at 160 kDa), eNOS (at 140 kDa) or IkB-α (at 40 kDa). and subsequently with appropriate secondary antibodies conjugated to horseradish peroxidase (Amersham, Buckinghamshire, UK). Immunoreactive bands were detected with SuperSignal chemiluminescent substrate (Pierce, Cheshire, UK) using Kodak autoradiography film (G.R.I., Rayne, UK). To ensure equal loading of proteins, β-actin levels were detected using primary rabbit anti-humanβ-actin antibody followed by the secondary anti-rabbit IgG horse-radish peroxidase conjugated antibody (Cell Signaling, USA). Images were finally analysed and quantified by densitometry essentially as described [5] and all band intensity data were normalized to β-actin levels and expressed as ratios to the relevant control (assigned a value of 1).

### VSMC studies

Primary rat aortic smooth muscle cell (VSMC) cultures were obtained by enzymatic dissociation of the thoracic aortas taken from untreated male Wistar rats and characterized as smooth muscle cells by morphology (multilayer sheets, ‘hills and valleys’) and immunostaining with monoclonal antibody specific for smooth muscle α-actin as described by us previously [4]. Cells were passaged upon reaching confluence with 0.5% trypsin-containing 0.2% EDTA and utilized between passages 3 and 10. For the mechanistic studies, VSMCs were initially cultured in serum containing DMEM media until 60–70% confluence and then in serum-free DMEM media containing either normal (5.5 mM) or high (25 mM) D-glucose and/or co-treated with different doses of the named drugs for 72 h. Cells were then lysed in cell lysis buffer, and total proteins were estimated and equivalent amounts of proteins were subjected to SDS-PAGE and immunoblotting as described above.

### Statistical analysis

Results were analyzed using Graph-pad Prism software. Data are presented as Mean ± SEM. of ‘N’ number of experiments. Mean values were compared using two-way analysis of variance followed by post hoc test (Bonferroni). The difference was considered to be significant when p value was less than 0.05.

## Results

### Hyperglycemia and Animals’ body weights

After four weeks of diabetes blood glucose levels were 590±26 mg/dl in STZ-induced diabetic animals as compared with 83±16 mg/dl in the non-diabetic control animals. Treatment with AG825 (585±17 mg/dl) or Ang-(1–7) [575±20 mg/dl] treatment did not significantly reduce blood glucose levels. There was a significant reduction of around 80g in the weights of STZ-diabetic rats (175±6 g) compared to the non-diabetic control animals (255±4g). Chronic Ang-(1–7), but not AG825, treatment for 4 weeks significantly improved the weight of diabetic rats to 199 ±9g and 185 ±9g, respectively (P<0.05).

### Chronic (in vivo) Ang-1-7 treatment inhibits diabetes-induced transactivation of ErbB2 receptor in the isolated mesenteric vascular bed

**Fig 1:**
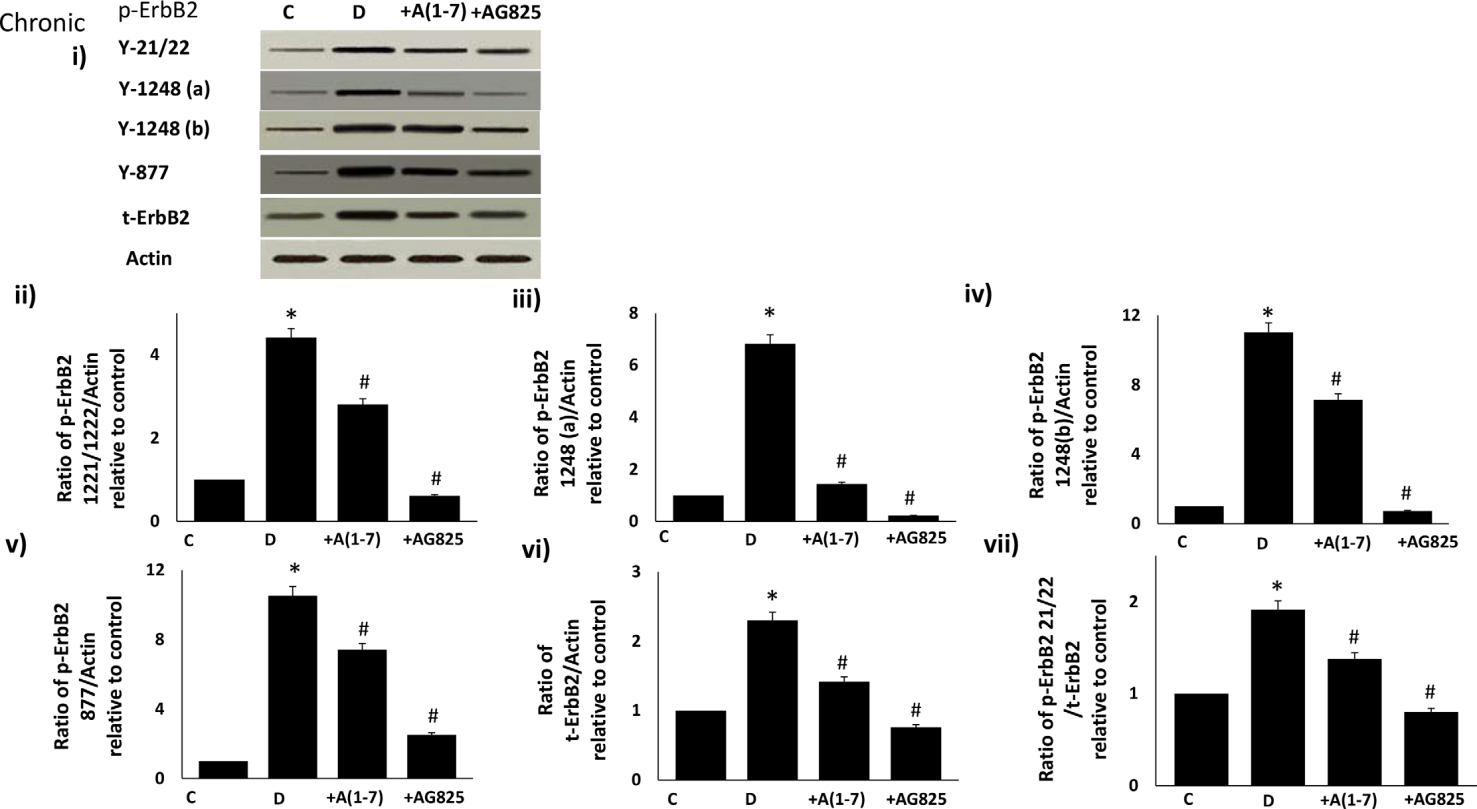
Diabetes-induced phosphorylation of ErbB2 receptor at multiple tyrosine residues can be attenuated by chronic treatment with Ang-(1–7) or AG825 in the mesenteric bed vasculature of STZ-induced diabetic rats. Panel i) is a representative Western blot showing the levels of phosphorylated ErbB2 receptor (p-ErbB2) at the indicated tyrosines Y1221/1222, Y1248 (detected by two separate antibodies labeled as Y1248a and Y1248b), and Y877, total ErbB2 receptor (t-ErbB2) and β-actin in the isolated mesenteric bed from normal (non-diabetic) controls (C), diabetic (D) and diabetic animals treated for 4 weeks with either Ang-(1–7) (+A1-7) or AG825 (+AG825). Panels ii)-vii) are densitometry histograms showing band intensity ratios for levels of phosphorylated (at the stated tyrosine residue) and total ErbB2 receptor normalized to actin and presented as relative to control (ii-vi) and ratios of phosphorylated ErbB2 (Y1221/1222) receptor to total ErbB2 receptor following normalization of each to actin and presented as relative to control value (panel vii). n = 6; mean ± SD. Asterisk (*) indicates significantly different (P < 0.05) mean values from normal non-diabetic rats (C), whereas # indicates significantly different mean values (P < 0.05) from diabetic rats (D).

Diabetes-induced phosphorylation of ErbB2 receptor was significantly attenuated by chronic treatment with Ang (1–7) or AG825, a selective inhibitor of ErbB2 receptor, at multiple tyrosine residues Y1221/1222, Y1248 (detected by two separate antibodies and labeled as Y1248a or Y1248b) and Y877 in the mesenteric vascular bed of STZ-induced diabetic rats (Fig 1 panels i-vii). Diabetes also partly increased expression of ErbB2 receptor protein that could be attenuated by Ang-(1–7) and AG825 treatment (Fig 1vi). However, Ang-(1–7), similar to AG825, exhibited a net inhibitory effect on phosphorylation of ErbB2 receptor as highlighted in ratio plot of the Y1221/22 phosphorylation site relative to total ErbB2 protein (Fig 1vii).

### Chronic or acute Ang-1-7 treatment opposes diabetes-induced changes in downstream signaling via ERK1/2, p38 MAPK, ROCK, eNOS and IkB-α signaling

**Fig 2:**
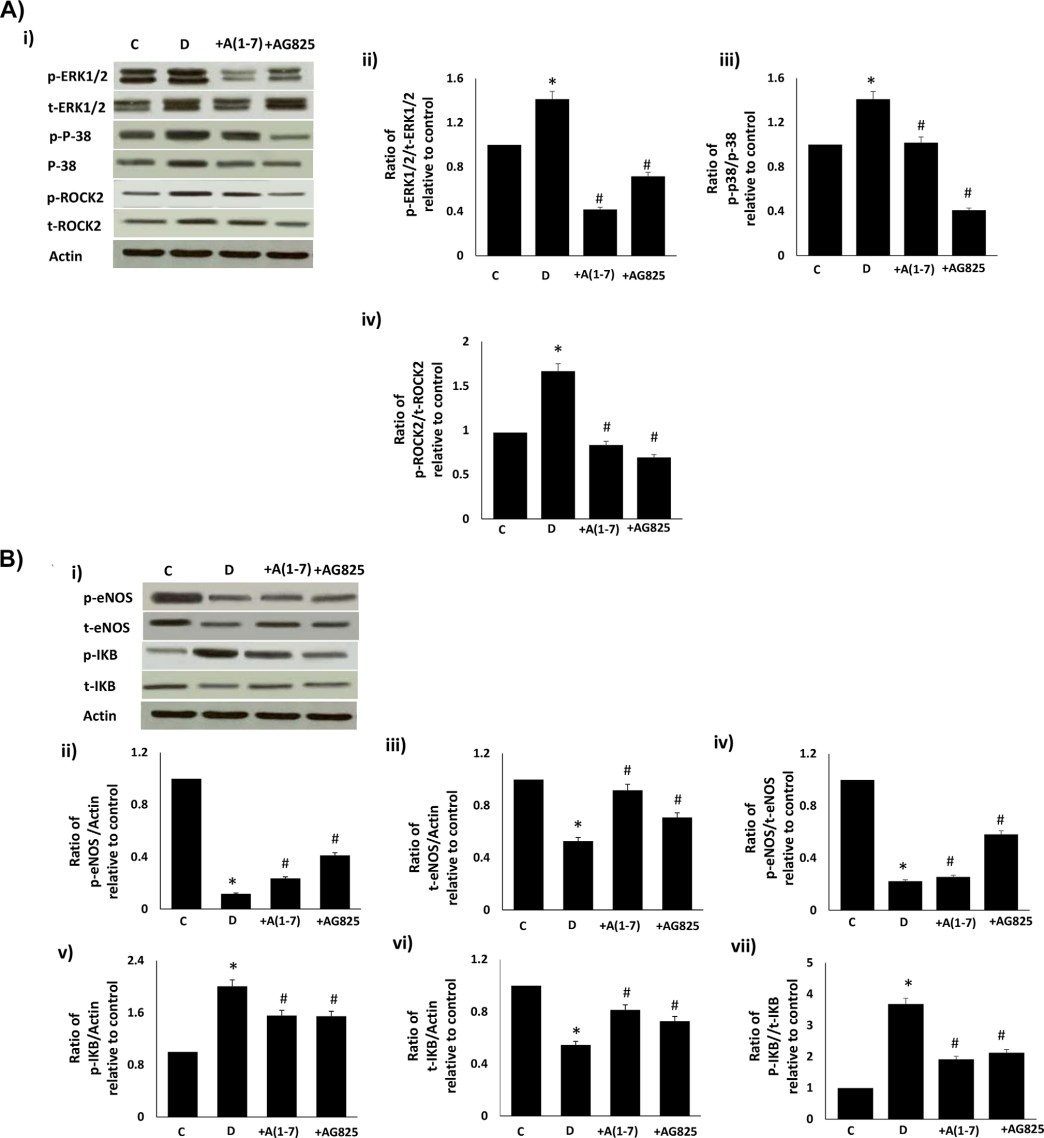
Chronic Ang-1-7 treatment opposes diabetes-induced signaling changes in (a) ERK1/2, p38 MAPK, ROCK2 and (b) in eNOS and NF-kB in the mesenteric vascular bed. **(a)** Representative Western blot (panel i) showing the levels of phosphorylated (p-) or total (t) for ERK1/2, p38 MAPK, ROCK2 proteins and β-actin in the isolated mesenteric bed from normal controls (C), diabetic (D) and diabetic animals treated for 4 weeks with Ang-(1–7) (+A1-7) or AG825 (+AG825). Densitometry histograms showing band intensity ratios for levels of phosphorylated to total protein as stated following normalization of each to actin and presented as relative to control value (panels ii-iv). (b) Representative Western blot (panel i) showing the levels of phosphorylated (p-) or total (t) for eNOS and IkB proteins and β-actin in the isolated mesenteric bed from normal controls (C), diabetic (D) and diabetic animals treated for 4 weeks with Ang-(1–7) (+A1-7) or AG825 (+AG825). Densitometry histograms showing band intensity ratios for levels of phosphorylated (p-) or total (t) proteins normalized to actin and presented as relative to control or for ratio of phosphorylated to total protein as stated following normalization to actin and presented as relative to control value (ii-vii). n = 6; mean ± SD. *Indicates significantly different (P < 0.05) mean values from normal non-diabetic rats (C), whereas # indicates significantly different mean values (P < 0.05) from diabetic rats (D).

**Fig 3:**
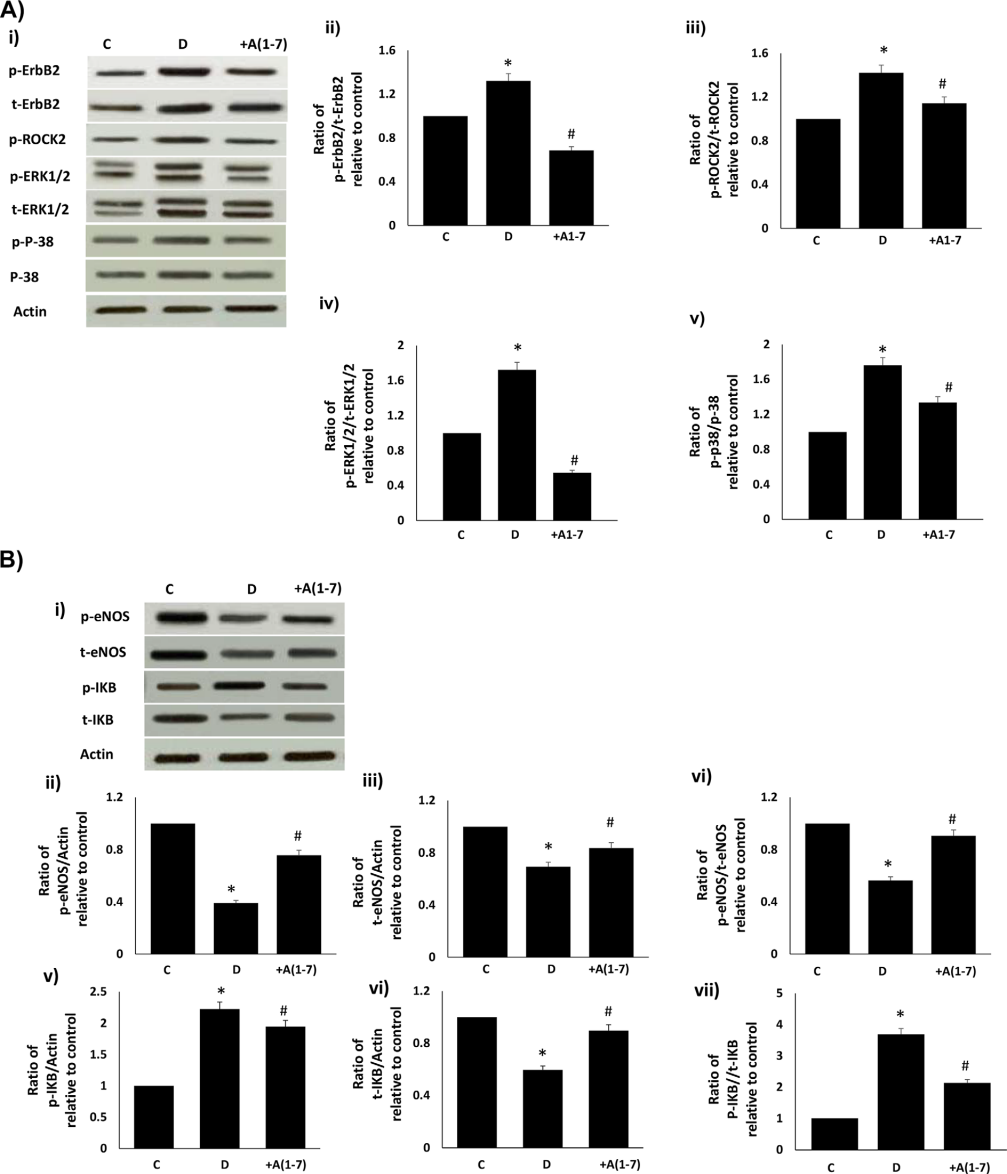
Acute (ex-vivo) Ang-1-7 treatment opposes diabetes-induced signaling changes in (a) ErbB2, ROCK2, ERK1/2, p38 MAPK and (b) in eNOS and NF-kB in the isolated mesenteric vascular bed. **(a)** Representative Western blot (i) showing the levels of phosphorylated (p-) or total (t) for ErbB2, ROCK2, ERK1/2, p38 MAPK proteins andβ-actin in the isolated mesenteric bed from normal controls (C), diabetic (D) and diabetic animals treated for 4 weeks with Ang-(1–7) (+A1-7). Densitometry histograms showing band intensity ratios for levels of phosphorylated (p-) to total (t) as stated following normalization of each to actin and presented as relative to control (ii-v). **(b)** Representative Western blot (i) showing the levels of phosphorylated (p-) or total (t) for eNOS and IkB proteins and β-actin in the isolated mesenteric bed from normal controls (C), diabetic (D) and diabetic animals treated for 4 weeks with Ang-(1–7) (+A1-7) or AG825 (+AG825). Densitometry histograms showing band intensity ratios for levels of normalized phosphorylated (p-) or total (t) proteins presented as relative to control or for ratio of phosphorylated to total protein as stated following normalization of each to actin and presented as relative to control (ii-vii). n = 6; mean ± SD. Asterisk (*) indicates significantly different (P < 0.05) mean values from normal non-diabetic rats (C), # indicates significantly different mean values (P < 0.05) from diabetic rats (D) and $ indicates significantly different mean values (P < 0.05) from Ang-(1–7).

Chronic in vivo administration of Ang-(1–7) or AG825 attenuated the diabetes-induced increases in phosphorylated and total ERK1/2, p-38 MAP kinase and ROCK2 (Fig 2a). Further, chronic Ang-(1–7) or AG825 treatment opposed the diabetes-induced reduction in total or phosphorylated (Ser1177) eNOS, elevation in phosphorylated IkB-α (a commonly used marker for NF-kB activity) and reduction in total eNOS in the mesenteric vascular bed (Fig 2b). These diabetes-induced changes in the net phosphorylation of these signaling molecules bed could also be significantly reversed by acute, ex vivo treatment of the isolated mesenteric vascular bed with Ang-(1–7) (Fig 3a and 3b).

### Ang-(1–7) inhibits high glucose-induced transactivation of ErbB2 receptor and downstream ERK1/2 signaling in VSMC

**Fig 4:**
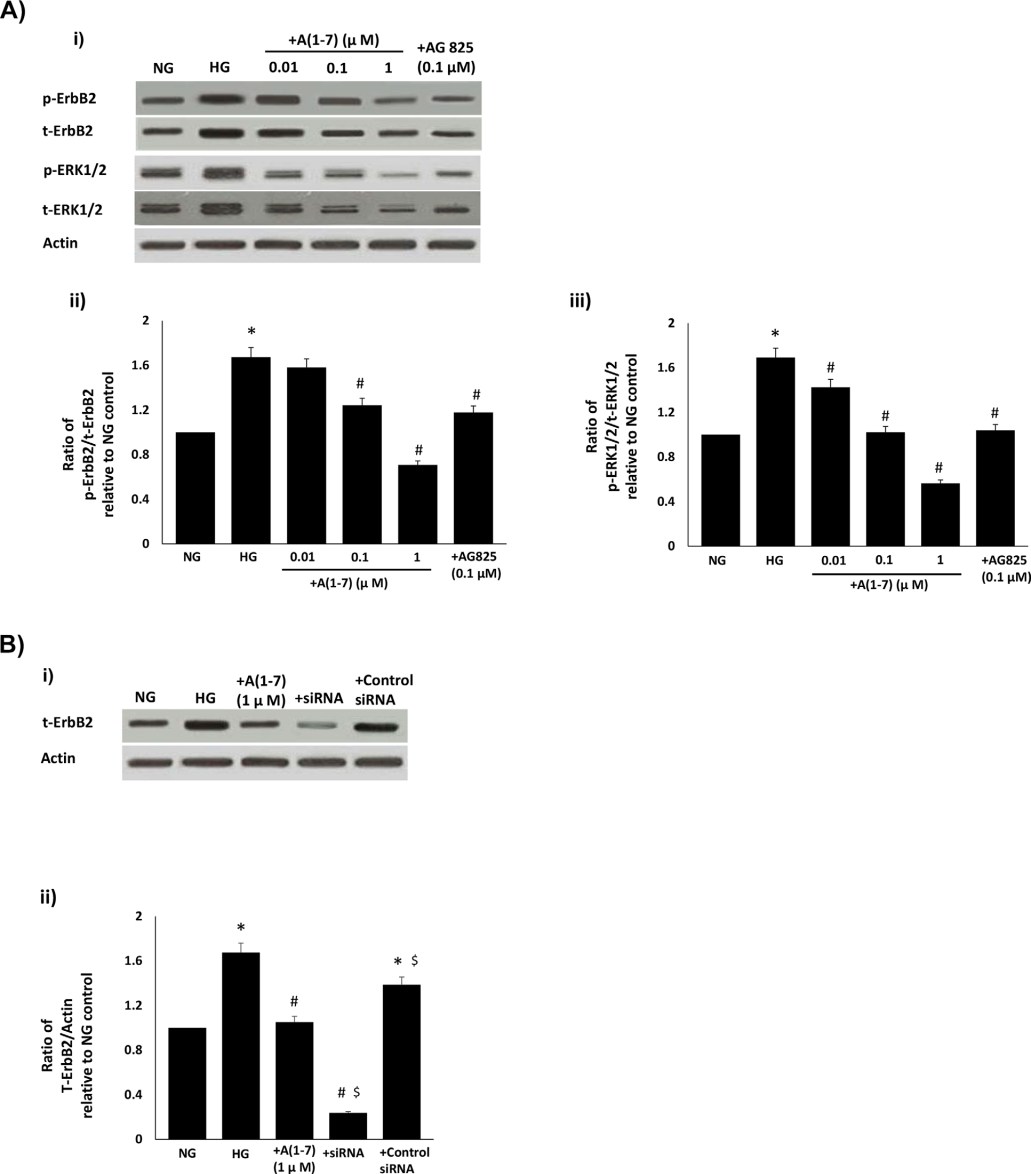
The effects of Ang-(1–7) on a) high glucose-induced transactivation of ErbB2 receptor at Y1221/1222 and of downstream ERK1/2 signaling and b) ErbB2 receptor expression when compared with anti-ErbB2 siRNA in VSMC. **(a)** Representative Western blot (panel i) showing the levels of phosphorylated (p-) or total (t) ErbB2, or ERK1/2 and total β-actin in VSMC grown in normal (5.5mM) D-glucose (NG) or cultured for 72h in high glucose (25mM D-glucose; HG) or HG co-treated with increasing (0.01, 0.1 and 1 micromolar doses of Ang-(1–7) (+ A(1–7)) or with AG825 (0.1 micromolar). Panels ii-iii) are densitometry histograms showing band intensity ratios for levels of phosphorylated (p-) to total (t) proteins as stated following normalization of each to actin and presented as relative to NG control. N = 5; Mean ± SD. Asterisk (*) indicates significantly different (p<0.05) mean values from NG whereas hash (#) indicates significantly different mean values (p<0.05) from HG; **(b)** Upper panel shows a representative Western blot showing the levels of phosphorylated (p-) or total (t) ErbB2 and total β-actin in VSMC grown in normal (5.5mM) D-glucose (NG) or cultured for 72h in high glucose (25mM D-glucose; HG) or HG co-treated with 1 micromolar Ang-(1–7) (+ A(1–7)) or with a pool of anti-ErbB2 siRNA or control siRNA (at 25 nanomolar concentrations). Lower panel shows the densitometry histograms showing band intensity levels of total (t) ErbB2 receptor protein normalized to actin and presented relative to NG control. N = 4; mean ± SD. Asterisk (*) indicates significantly different (p<0.05) mean values from NG whereas hash (#) indicates significantly different mean values (p<0.05) from HG and $ indicates significantly different to +A(1–7) treatment.

We next studied the effects of Ang-(1–7) treatment on high glucose-induced ErbB signaling in primary VSMC obtained from non-diabetic rats and cultured in high glucose (25mM)—a model we have previously shown to effectively mirror signaling changes in the diabetic mesenteric vascular bed [4,5]. Ang-(1–7) treatment inhibited high glucose-induced increases in total and phosphorylated levels of ErbB2 (Y1221/22) and ERK1/2 in a dose-dependent manner in VSMC (Fig 4a, panel i). However, Ang-(1–7) had a net inhibitory effect on the phosphorylation of ErbB2 and ERK1/2, in a manner similar to that observed for AG825 (p<0.05; Fig 4a, ii–iii). Further, Ang-(1–7)-mediated inhibition of high glucose-induced total ErbB2 protein expression was less marked than that obtained by gene silencing with anti-ErbB2 siRNA (Fig 4b).

### Ang-(1–7) inhibits high glucose-induced ErbB2 receptor transactivation by inhibiting Src phosphorylation via a Mas receptor-dependent mechanism in VSMC

**Fig 5:**
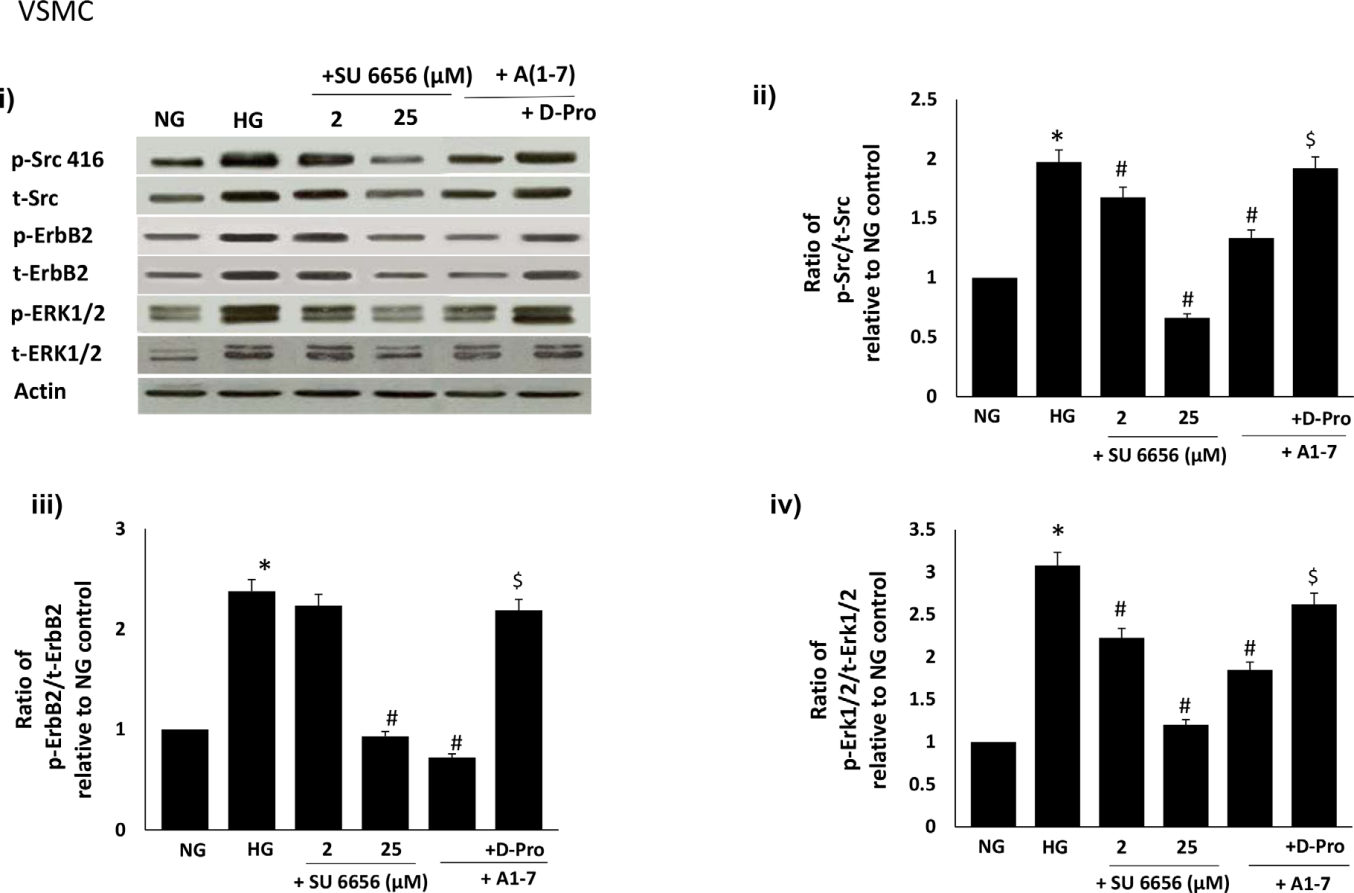
High Glucose-induced ErbB2-ERK1/2 phosphorylation occurs via a src-dependent pathway that can be blocked by Ang-(1–7) via its Mas receptor. Panel (i) is a representative Western blot showing the levels of phosphorylated Src at Y416 (p-Src), total Src (Src), phosphorylated ErbB2 receptor at Y1221/1222 (p-ErbB2), total ErbB2 receptor (t-ErbB2) and phosphorylated (p-) and total (t-) ERK1/2 in VSMC grown in normal (5.5 mM) D-glucose (NG), high glucose (25 mM) D-glucose (HG) or HG treated with increasing doses (2 and 25 micromolar) of Src selective inhibitor, SU6656 (lanes labelled as + SU6656 and the stated dose) or HG treated with either 1 micromolar Ang-(1–7) (+ A(1–7) or together with the selective Mas receptor inhibitor, D-Pro^7^-Ang-(1–7) (lane labeled as + D-Pro). Panels (ii-vii) are densitometry histograms showing ratio levels of phosphorylated to total proteins as stated following normalization of each to actin and presented relative to NG control. N = 5; Mean ± SD. Asterisk (*) indicates significantly different (p<0.05) mean values from NG whereas hash (#) indicates significantly different mean values (p<0.05) from HG.

We next showed that high glucose-induced phosphorylation of Src (Y416) was attenuated in a dose-dependent manner by the selective Src inhibitor, SU6656 in VSMC (see Fig 5). Inhibition of glucose-induced Src phosphorylation at Y416 by SU6656 also led to a dose-dependent inhibition of ErbB2 receptor and downstream ERK1/2 phosphorylation in VSMC (Fig 5). High glucose-induced Src phosphorylation and subsequent signalling via the ErB2receptor/ ERK1/2 pathway could be inhibited by Ang-(1–7) (p<0.05; Fig 5). The inhibitory effect of Ang-(1–7) on Src/ErbB2 receptor/ERK1/2 signalling could be significantly reversed by the selective Mas receptor inhibitor [D-Pro^7^-Angiotensin- (1–7)] (p<0.05; Fig 5b).

### Ang-(1–7) inhibits Ang II-mediated transactivation of ErbB2 receptor in VSMC

**Fig 6:**
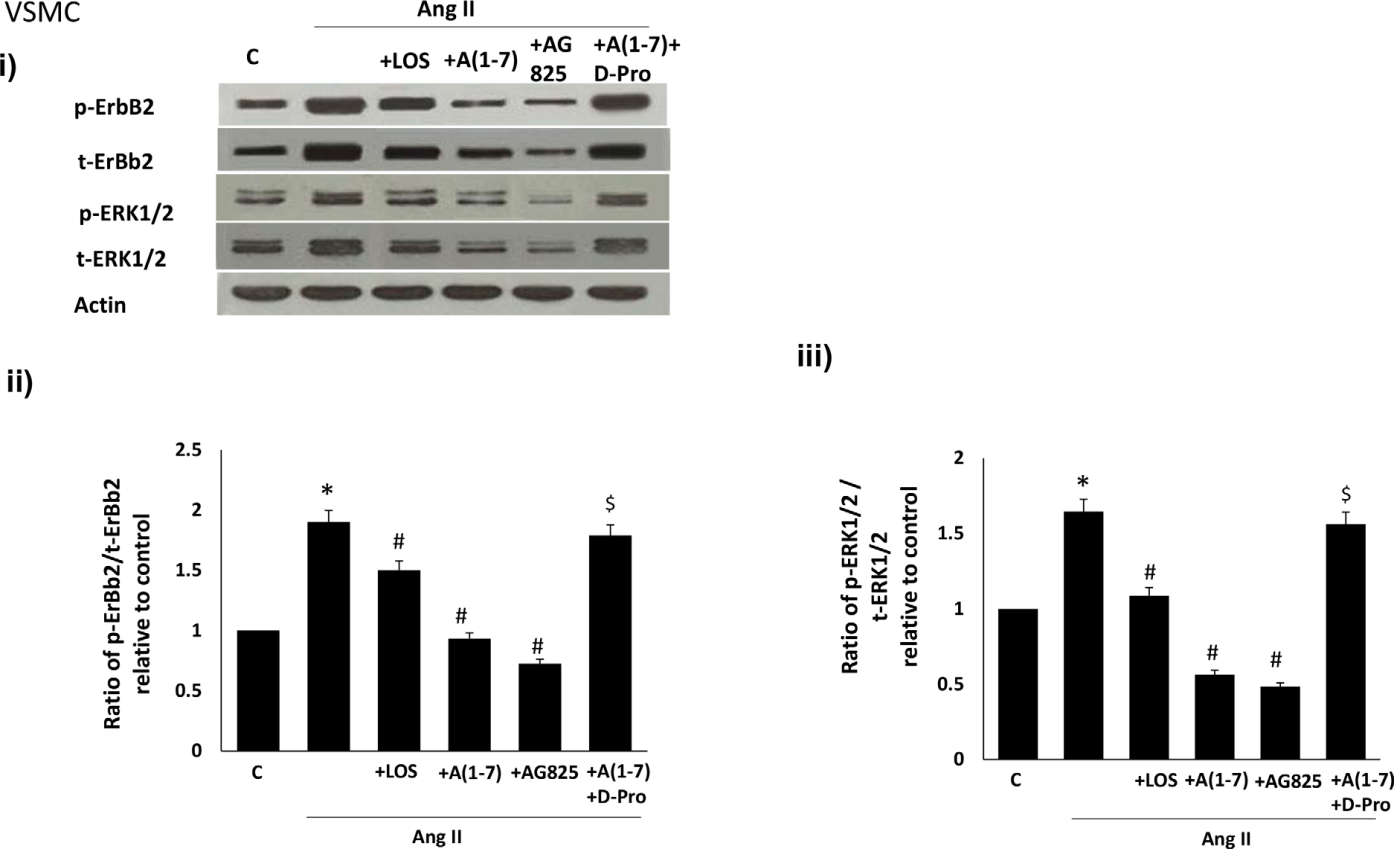
Ang-(1–7) via its Mas receptor inhibits Ang II-mediated transactivation of ErbB2 receptor and downstream ERK1/2 phosphorylation in VSMC in a manner similar to that of an AT_1_ receptor antagonist, losartan, and AG825, a selective inhibitor of ErbB2 receptor phosphorylation. (i) Representative Western blot showing the levels of phosphorylated (p-) or total (t-) ErbB2 receptor (Y1221/1222), or ERK1/2 and total βa-actin in VSMC grown in normal (5.5 mM) D-glucose (NG), or treated with 1 micromolar Ang II (+Ang II), or together with 1 micromolar losartan (+LOS), with 0.1 micromolar AG825 (+AG825) or 1 micromolar Ang-(1–7) (labelled as A(1–7) or together with the selective Mas receptor inhibitor, D-Pro^7^-Ang-(1–7) (lane labeled as A(1–7) + D-Pro); Panels ii-iii are densitometry histograms showing ratio levels of phosphorylated to total proteins as stated following normalization of each to actin and presented relative to control. N = 5; Mean ± SD. Asterisk (*) indicates significantly different (p<0.05) mean values from NG whereas hash (#) indicates significantly different mean values (p<0.05) from HG.

We next studied the effects of Ang-(1–7) on Ang II-mediated transactivation of ErbB2 in VSMC. Ang II led to transactivation of ErbB2 receptor as evidenced by an enhanced phosphorylation at Y1221/22 as well as enhanced phosphorylation of ERK1/2 in VSMC (Fig 6). Ang-(1–7) inhibited Ang II-mediated phosphorylation of ErbB2 receptor and ERK1/2 in a manner similar to that of Losartan, an Ang II type 1 receptor (AT1) receptor blocker, or AG825 (Fig 6). The inhibitory effect of Ang-(1–7) could be significantly reversed by the selective Mas receptor antagonist, [D-Pro^7^-Angiotensin- (1–7)] (p<0.05; Fig 6).

### Ang-(1–7) inhibits NE-mediated transactivation of ErbB2 and EGFR receptor via its Mas receptor in VSMC

**Fig 7:**
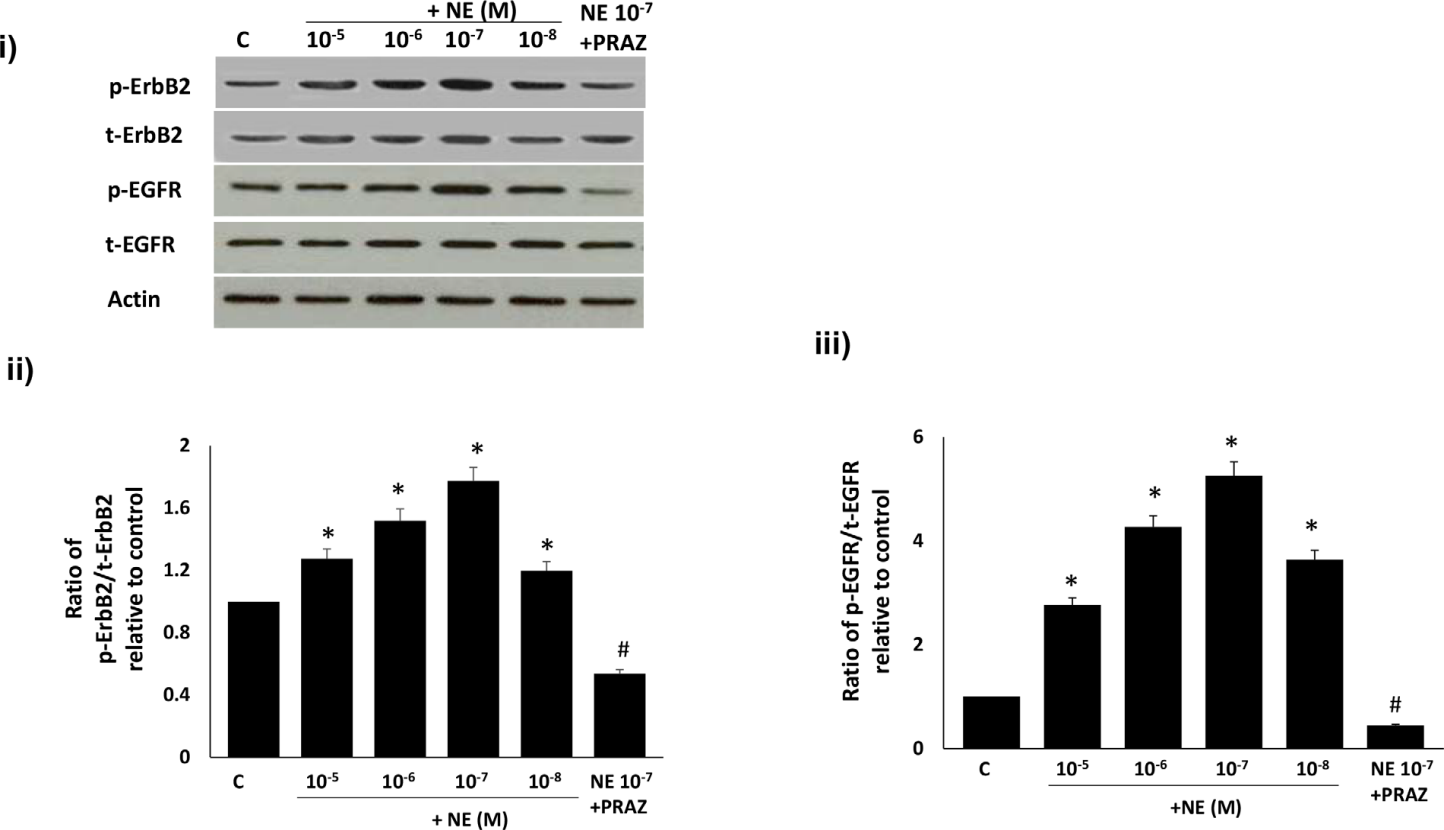
The dose-dependent transactivation of ErbB2 and EGFR receptor by norepinephrine in VSMC can be attenuated by the α_1-_adrenergic receptor inhibitor, Prazosin. Panel (i) is a representative Western blot showing the levels of total (t-) or phosphorylated (p-) ErbB2 receptor (Y1221/1222), EGFR (Y1068) and total β-actin in VSMC grown in normal (5.5 mM) D-glucose (NG), or NG treated with increasing doses of NE or NE (10^-7^M) together with 1 micromolar Prazosin (labelled as NE 10^−7^ + PRAZ); Panels ii-iii are densitometry histograms showing ratio levels of phosphorylated to total proteins as stated following normalization of each to actin and presented relative to control. N = 5; Mean ± SD. Asterisk (*) indicates significantly different (p<0.05) mean values from NG whereas hash (#) indicates significantly different mean values (p<0.05) from NG at 10^-7^M dose.

**Fig 8:**
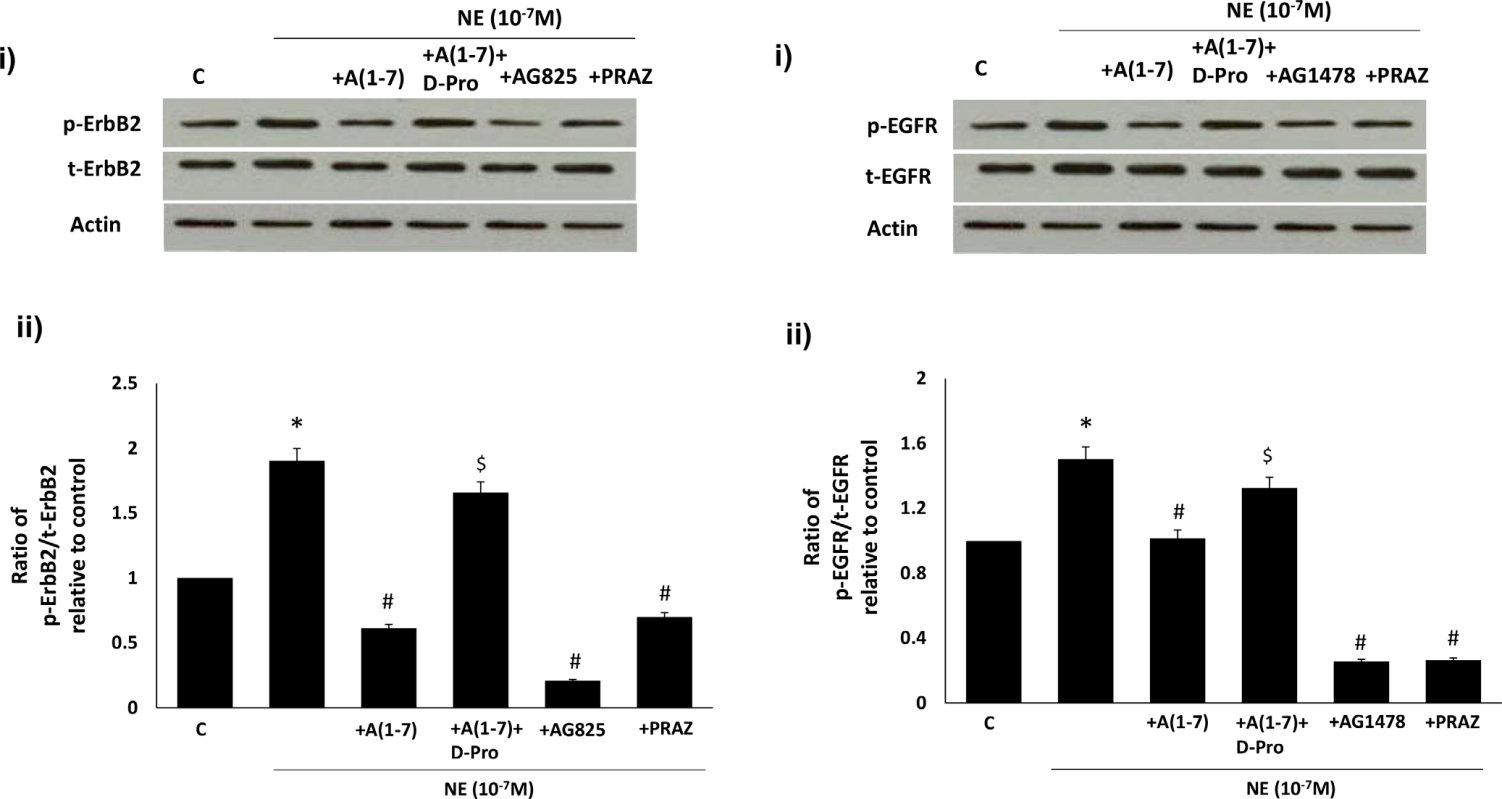
Ang-(1–7) via its Mas receptor inhibits NE-mediated transactivation of (A) ErbB2 and (B) EGFR receptors in VSMC. In both A) and B) panel (i) is a representative Western blot showing either the levels of total (t-) or phosphorylated (p-) ErbB2 receptor (Y1221/1222) or EGFR (Y1068) and total β-actin in VSMC grown in normal (5.5 mM) D-glucose (NG), or NG treated with NE (10^-7^M) or NE together with 1 micromolar Ang-(1–7) (+ A(1–7), or A(1–7) together with D-Pro^7^-Ang-(1–7) (lane labeled as + A(1–7) +D-Pro), AG1478 (1μM) or AG825 (0.1μM) or 1 micromolar Prazosin (labelled as NE 10^−7^ + PRAZ); Panels ii is the densitometry histogram showing ratio levels of stated phosphorylated to total proteins following normalization of each to actin and presented relative to control. N = 5; Mean ± SD. Asterisk (*) indicates significantly different (p<0.05) mean values from NG whereas hash (#) indicates significantly different mean values (p<0.05) from NG at 10^-7^M dose.

We next compared the effects of Ang-(1–7) on NE-mediated transactivation of ErbB2 and EGFR in VSMC. A dose dependent NE-mediated increase in phosphorylation of both ErbB2 and EGFR receptors was observed that was followed by a decrease at higher doses (Fig 7). At the optimal stimulatory concentration of NE (10^-7^M) for both receptors, NE-induced phosphorylation of ErbB2 and EGFR was blocked by the selective α-_1_-adrenoceptor antagonist, Prazosin (p<0.05; Fig 7). Furthermore, Ang-(1–7) significantly inhibited NE-mediated phosphorylation of both ErbB2 and EGFR receptors that could be significantly reversed by the selective Mas receptor antagonist, [D-Pro^7^Angiotensin- (1–7)]. In addition, NE-mediated phosphorylation of ErbB2 and EGFR receptors could be significantly attenuated by AG825 and AG1478 respectively as well as by Prazosin (p<0.05; Fig 8).

### Ang-(1–7) inhibits diabetes- and high glucose-induced transactivation of ErbB3 and ErbB4 receptors

**Fig 9:**
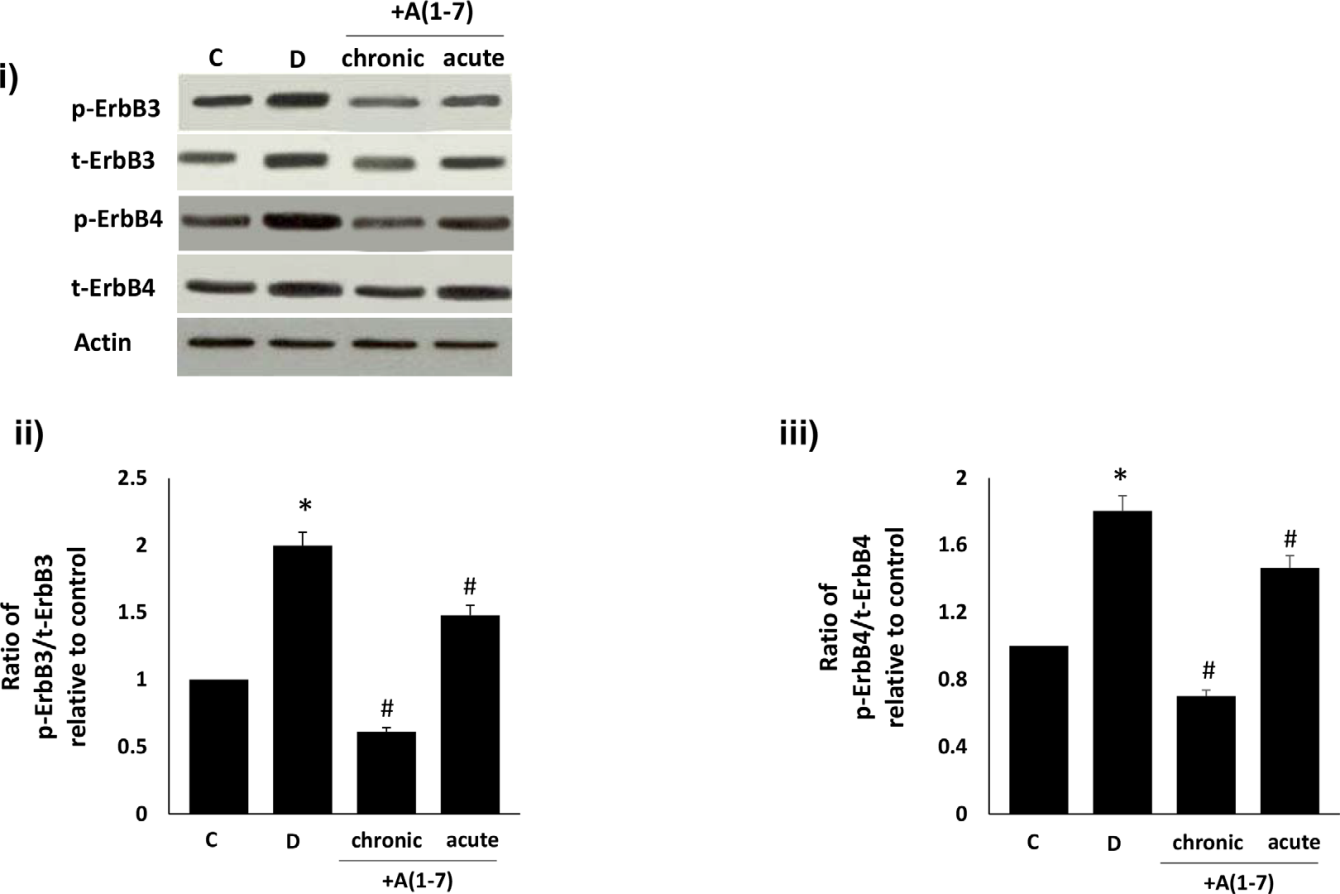
Chronic (in vivo) and acute (ex vivo) Ang-(1–7) treatment inhibits diabetes-induced transactivation of ErbB3 and ErbB4 receptors in the isolated mesenteric vascular bed. Panel (i) is a representative Western blot showing the levels of total (t-) or phosphorylated (p-) ErbB3 (Y1222), ErbB4 (Y1284) receptors and total β-actin in the isolated mesenteric bed from normal controls (C), diabetic (D) and diabetic animals treated chronically for 4 weeks (lane labeled as chronic) or acutely ex vivo with 1 micromolar (lane labeled as acute) Ang-(1–7) (+A1-7). Panels ii-iii) are densitometry histograms showing ratio levels of stated phosphorylated (p-) to total (t) proteins following normalization of each to actin and presented relative to control. N = 6; mean ± SD. Asterisk (*) indicates significantly different (P < 0.05) mean values from normal non-diabetic rats (C), whereas # indicates significantly different mean values (P < 0.05) from diabetic rats (D).

**Fig 10:**
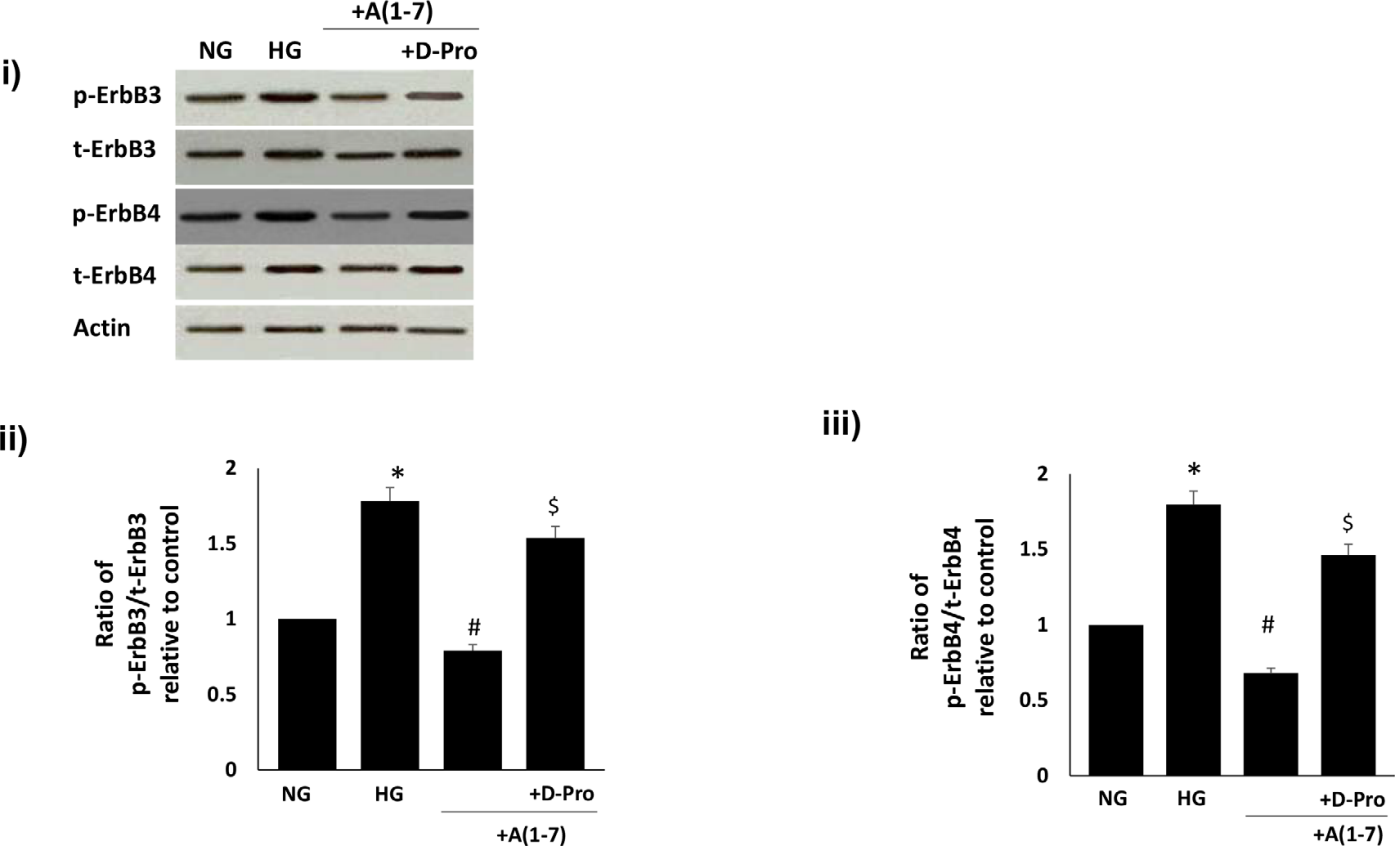
Ang-(1–7) via its Mas receptor inhibits high glucose-induced transactivation of ErbB3 and ErbB4 receptors in VSMC. Panel (i) is a representative Western blot showing the levels of total (t-) or phosphorylated (p-) ErbB3 (Y1222), ErbB4 (Y1284) receptors and total β-actin in VSMC grown in normal (5.5 mM) D-glucose (NG), or treated with 1 micromolar Ang-(1–7) (labelled as A1-7) or A(1–7) together with D-Pro^7^-Ang-(1–7) (+D-Pro). Panels ii-iii are densitometry histograms showing ratio levels of stated phosphorylated to total protein following normalization of each to actin and presented relative to control. N = 5; Mean ± SD. Asterisk (*) indicates significantly different (p<0.05) mean values from NG whereas hash (#) indicates significantly different mean values (p<0.05) from HG.

We next investigated the effects of diabetes or high glucose and Ang-(1–7) treatment on ErbB3 and ErbB4 receptor phosphorylation in vivo and in vitro. Diabetes resulted in significantly enhanced total and phosphorylated forms of ErbB3 and ErbB4 receptors that could be significantly reversed by chronic in vivo and, to a lesser extent, by acute ex vivo treatment with Ang-(1–7) in the diabetic mesenteric vascular bed (p<0.05; Fig 9). Furthermore, Ang-(1–7) also attenuated high-glucose induced increases in total and phosphorylated forms of ErbB3 and ErbB4 receptors in VSMC that could be significantly attenuated by the selective Mas receptor antagonist, [D-Pro^7^-Angiotensin- (1–7)] (p<0.05; Fig 10). Although increased levels of total ErbB3 and ErbB4 receptor proteins were noted in the diabetic state and in VSMC cultured in high glucose for 72h, there was a net increase in phosphorylation as indicated by the ratio plots of phosphorylated to total proteins (Figs 9ii and 9iii, 10ii and 10iii). Although Ang-(1–7) treatment in vivo or in VSMC partly inhibited ErbB3 and ErbB4 receptor expression (9i and 10i), it appeared to have a net inhibitory effect on ErbB3 and ErbB4 receptor phosphorylation (Figs 9ii and 9iii, 10ii and 10iii).

## Discussion

This study reports on several novel findings: a) the major finding is that Ang-(1–7) is an inhibitor of all the ErbB family of receptor tyrosine kinases, b) high glucose-mediated transactivation of ErbB2 and downstream phosphorylation of ERK1/2 occurs via a src-dependent pathway and that Ang-(1–7) via its Mas receptor blocks this pathway, c) p38 MAP kinase, NF-kB and eNOS appear to be downstream effectors of ErbB2 receptor as their diabetes-induced alterations can be reversed by AG825, a selective ErbB2 receptor antagonist as well as Ang-(1–7), d) AngII, as well as NE, induced transactivation of ErbB2 and/or EGFR that could be blocked by Ang-(1–7) via its Mas receptor in VSMC and e) diabetes or high glucose leads to transactivation of ErbB3 and ErbB4 receptors which, in the case of high glucose, could be blocked by Ang-(1–7) via a Mas receptor-dependent mechanism. These studies provide a novel insight into the actions of Ang-(1–7) in the vasculature and suggest for the first time that this heptatpeptide via its Mas receptor acts as a global- or pan-inhibitor of ErbB receptor transactivation.

We previously showed that the dysregulation of ErbB receptor tyrosine kinases appears to be an important early initiation step in the development of diabetes-induced vascular dysfunction that involves increased gene-expression and enhanced phosphorylation of EGFR and ErbB2 receptor tyrosine kinases [4–5, 7–9]. Receptor tyrosine kinase inhibitors of EGFR or ErbB2 as well as Ang-(1–7) appear to be effective treatments for diabetes-induced vascular dysfunction in animal models [4–5, 7–9, 32]. We had previously shown that these beneficial actions of Ang-(1–7) may involve inhibition of EGFR transactivation [5] but its effects on the transactivation of ErbB2 as well as other ErbB receptor family members was not known and thus studied here.

At a dose and treatment regimen previously shown to correct diabetes-induced vascular dysfunction, we firstly showed that chronic in vivo or acute (ex vivo) Ang-(1–7) treatment can inhibit diabetes-induced transactivation of ErbB2 receptor at multiple tyrosine sites (namely Y1221/1222, Y1248 and Y877) and correct changes in downstream signaling molecules (ERK1/2, p-38 MAPK, ROCK2, eNOS and IkB-α) within the mesenteric vascular bed without correcting the underlying hyperglycemia. The enhanced phosphorylation of ErbB2 in the vasculature and the subsequent effects of Ang-(1–7), or indeed AG825, could only partly be explained by a change in receptor expression. It is clear that Ang-(1–7) can partly inhibit diabetes- or high glucose-mediated elevation in ErbB2 protein expression, though not as marked as that achieved by siRNA in VSMC, whereas its effects on ErbB2 receptor phosphorylation appear greater. This was more obvious in the case of acute ex vivo studies where a marked reduction in ErbB2 phosphorylation, with little change in total receptor protein, was noted upon Ang-(1–7) treatment compared to diabetes alone (Fig 3a panel i). These data collectively imply that there is a net increased phosphorylation of ErbB2 in the diabetic mesenteric vascular bed, similar to that reported for EGFR [4] and that Ang-(1–7) has a net effect on ErbB2 phosphorylation similar to that observed for AG825 (Fig 1 panel i).

We had previously shown that diabetes-induced elevation in ErbB2 and subsequent downstream signaling via ROCK and ERK1/2 were involved in mediating vascular dysfunction in the mesenteric vascular bed [4]. The fact that in the present study selective inhibition of ErbB2 with AG825 also led to inhibition of p-38 MAPK, eNOS and IkB-α suggests that these signaling molecules also represent downstream signaling pathways for ErbB2 signaling in the diabetic vasculature. Although it has been previously shown that Ang-(1–7) can inhibit eNOS and IkB/NF-kB signaling pathways [33–38], the present study suggests that, at least in the case of diabetes-induced vascular dysfunction, this might arise from upstream inhibition of ErbB2 by Ang-(1–7).

The precise mechanism by which diabetes and/or hyperglycemia transactivates ErbB2 receptors is not known. We provided evidence here that high glucose-mediated transactivation of ErbB2 and downstream ERK1/2 phosphorylation in VSMC occurs via phosphorylation of the non-receptor tyrosine kinase, Src, at Y416 (Fig 5). The selective c-src tyrosine kinase inhibitor, SU6656, led to a dose-dependent inhibition of high glucose-induced phosphorylation of Src (Y416) as well as ErbB2 (Y1221/1222) and ERK1/2 phosphorylation implying that Src phosphorylation was an upstream event of ErbB2-ERK1/2 signaling pathway. Further we showed that Ang-(1–7) inhibits ErbB2 transactivation and downstream signaling through ERK1/2 via inhibition of src phosphorylation in a manner similar to that described by us for EGFR [4–5]. The inhibitory effect of Ang-(1–7) on src-ErbB2-ERK1/2 phosphorylation could be reversed with the selective Mas receptor antagonist (D-Pro^7^-Ang-(1–7), implying that Ang-(1–7)-mediated inhibition of ErbB2 transactivation occurred via its Mas receptor. Our data showing Ang-(1–7) inhibiting Src phosphorylation are further supported by similar findings in cardiac fibroblasts [39] and in activated macrophages [40].

The fact that Ang-(1–7)/Mas receptor-mediated inhibition of ErbB2 transactivation also occurred in the presence of other potential triggers of ErbB2 receptor phosphorylation, namely Ang II and NE, implied that the inhibitory effects of Ang-(1–7) may be more general. In VSMC grown in normal glucose concentrations, Ang II-mediated ErbB2 transactivation occurred via the AT1 receptor as it could be blocked by Losartan- a selective AT1-receptor blocker. Ang-(1–7) significantly inhibited Ang II-mediated ErbB2 transactivation, in a manner similar to AG825, that could be effectively reversed by the Mas receptor antagonist (D-Pro^7^-Ang-(1–7), implying that its inhibition of Ang II-induced ErbB2 transactivation occurred via the Mas receptor. These data are consistent with the notion that ACE2/Ang-(1–7)/Mas receptor axis is beneficial via counter-regulating the ACE/AngII/AT1 receptor branch of the RAS which in the case of diabetes-induced vascular dysfunction exerts its detrimental actions through activation of ErbB receptors.

We also showed that NE can dose-dependently transactivate ErbB2 receptors at (Y1221/1222) as well as EGFR at Y1068 via its α_1_-adrenoceptors, as the NE-induced phosphorylation of these receptors could be significantly attenuated by Prazosin, an α_1_-adrenoceptor antagonist. We also showed that Ang-(1–7) inhibited NE-induced ErbB2 (Y1221/1222) and EGFR (Y1068) phosphorylation that could be reversed with the selective Mas receptor antagonist (D-Pro^7^-Ang-(1–7), implying that inhibition of α_1_-adrenoceptor-induced ErbB2 transactivation by Ang-(1–7) also occurred via its Mas receptor. Although NE-mediated transactivation of EGFR has been implicated in partly mediating the contraction of rat aorta [19], to the best of our knowledge this is the first report showing α_1_-adrenoceptor-mediated transactivation of ErbB2 receptors and that Ang-(1–7) via its Mas receptor is an effective blocker of this particular GPCR-mediated ErbB2 transactivation pathway. Thus, our data implies that α_1_-adrenoceptor blockade may be beneficial in the treatment of diabetes-induced vascular dysfunction. Since it is known that Ang-(1–7) levels are raised upon treatment with ACE inhibitors or AT_1_ receptor blockers and that they contribute to the beneficial effects in preventing diabetes-induced end-organ damage [24, 30], our data further leads us to speculate that Ang-(1–7) effects may be partly mediated via α_1_-adrenoceptor blockade of ErbB2 transactivation.

In contrast to EGFR and ErbB2 receptors [4–5,7–9, 31], the effect of diabetes or high glucose on the phosphorylation of other members of the ErbB family of receptor tyrosine kinases is not well characterized. Here, we showed that diabetes leads to enhanced phosphorylation of ErbB3 and ErbB4 receptors that can be blocked by chronic in vivo or acute ex vivo treatment with Ang-(1–7). Similarly, high glucose also induced a significant increase in ErbB3 and ErbB4 phosphorylation that could be blocked by Ang-(1–7). Since the effects of Ang-(1–7) on ErbB3 and ErbB4 phosphorylation could be reversed with the selective Mas receptor antagonist (D-Pro^7^-Ang-(1–7), this implied that high glucose-induced ErbB3 and ErbB4 transactivation, as well as ErbB2, could all be inhibited by Ang-(1–7) via its Mas receptor. Taken together with our previous data with EGFR [4–5], these results implicate Ang-(1–7) as a pan-inhibitor of ErbB receptor tyrosine kinase transactivation.

Further studies are needed to determine if Ang-(1–7) actions are specific to ErbBs or it acts as a general receptor tyrosine kinase inhibitor. The latter is unlikely as it actions on Insulin receptor signaling, another tyrosine kinase receptor, appear stimulatory [41–43]. Nonetheless, this study highlights the actions of Ang-(1–7)/Mas receptor in inhibiting ErbB receptors in the vasculature as a potential mechanism of action in the treatment of diabetes-induced vascular dysfunction. If the actions of Ang-(1–7) as a pan-ErbB inhibitor of receptor transactivation can be extended to other cell types beyond the vasculature described herein, then it might offer great potential in the treatment of other conditions where up-regulation of ErbB family of receptors is implicated. For example, there is great interest for the clinical development of pan-ErbB inhibitors in cancer where single receptor targeting has led to the development of resistance [44–46]. Although several small molecule pan-ErbB inhibitors are now in clinical trials evaluation for cancer therapy [46], our data suggests that Ang-(1–7), that also has significant anti-proliferative actions together with an acceptable safety profile in human clinical trials [47], might represent an attractive alternative treatment option for tumours reliant on ErbB receptors for growth and development.

Since ErbB2-4 receptors can heterodimerize with each other [12], further studies are also needed to determine if the actions of Ang-(1–7) involve interfering with ErbB receptor heterodimer formation, especially with EGFR as we have previously shown this receptor to be important in the development of diabetes-induced vascular dysfunction [7–9]. However, recent data from our laboratory [48] showed that specific inhibition of ErbB2 receptor with AG825 or siRNA had similar vascular effects as a dual EGFR/ErbB2 inhibitor implying that ErbB2 rather than EGFR may be the predominant signaling pathway involved in diabetes-induced vascular dysfunction. Thus, the inhibitory actions of Ang-(1–7) on ErbB2, as described in the present study, may be the critical step in its beneficial effects in reversing diabetes-induced vascular dysfunction; though this clearly needs further investigation.

In conclusion, this study has highlighted that diabetes and/or high glucose induced ErbB2 and downstream signaling pathways involving ROCK, p-38 MAP kinase, ERK1/2, eNOS, and IkB-α could be effectively blocked by Ang-(1–7) in vitro and in vivo. Ang-(1–7) acting through its Mas receptor could block ErbB2 transactivation induced by several triggers including high glucose, Ang II and NE in VSMC. The fact that high glucose and/or diabetes-induced phosphorylation of ErbB3 and ErbB4 could also be blocked by Ang-(1–7), leads us to conclude that at least in the diabetic vasculature Ang-(1–7) acts as a pan-ErbB inhibitor at the level of receptor transactivation. These finding may have important implications for the actions and potential use of Ang-(1–7) in treating diabetes-induced end organ damage as well as in cancer therapy.

## Abbreviations

AG1478: **N**-(3-chlorophenyl)-6,7-dimethoxy-4-quinazolinanine hydrochloride
AG825: ((E)-3-[3-[2-Benzothiazolythio)methyl]-4-hydroxy-5-methoxyphenyl]-2-cyano-2-propen-amide)
Ang I: angiotensin I
Ang II: angiotensin II
Ang (1–7): angiotensin (1–7)
DMEM: Dulbecco’s modified Eagle’s medium
EGF: epidermal growth factor
NE: (±) Norepinephrine/Noradrenaline-bitartrate
RAS: renin-angiotensin system
RTK: receptor tyrosine kinases
NF-kB: nuclear factor-kB
siRNA: small interfering RNA
STZ: streptozotocin
SU6656: 2,3-dihydro-N, N-dimethyl-2-oxo-3-[{4,5,6,7-tetrahydro-1**H**-indol-2-yl}methylene]-1**H**-indole-5-sulphonamide
VSMC: vascular smooth muscle cell
